# Transcriptome Analysis of *Thapsia laciniata* Rouy Provides Insights into Terpenoid Biosynthesis and Diversity in Apiaceae

**DOI:** 10.3390/ijms14059080

**Published:** 2013-04-25

**Authors:** Damian Paul Drew, Bjørn Dueholm, Corinna Weitzel, Ye Zhang, Christoph W. Sensen, Henrik Toft Simonsen

**Affiliations:** 1 Department of Plant and Environmental Sciences, Faculty of Sciences, University of Copenhagen, Frederiksberg DK-1871, Denmark; E-Mails: dpd@life.ku.dk (D.P.D.); due_bjoern@hotmail.com (B.D.); corinna.weitzel@web.de (C.W.); 2 Wine Science and Business, School of Agriculture Food and Wine, University of Adelaide, South Australia, SA 5064, Australia; 3 Department of Biochemistry and Molecular Biology, Faculty of Medicine, University of Calgary, Calgary, AB T2N 1N4, Canada; E-Mails: zhaye@ucalgary.ca (Y.Z.); csensen@ucalgary.ca (C.W.S.)

**Keywords:** *Thapsia laciniata*, guaianolides, thapsane, Apiaceae, sesquiterpenoids

## Abstract

*Thapsia laciniata* Rouy (Apiaceae) produces irregular and regular sesquiterpenoids with thapsane and guaiene carbon skeletons, as found in other Apiaceae species. A transcriptomic analysis utilizing Illumina next-generation sequencing enabled the identification of novel genes involved in the biosynthesis of terpenoids in *Thapsia*. From 66.78 million HQ paired-end reads obtained from *T. laciniata* roots, 64.58 million were assembled into 76,565 contigs (N50: 1261 bp). Seventeen contigs were annotated as terpene synthases and five of these were predicted to be sesquiterpene synthases. Of the 67 contigs annotated as cytochromes P450, 18 of these are part of the CYP71 clade that primarily performs hydroxylations of specialized metabolites. Three contigs annotated as aldehyde dehydrogenases grouped phylogenetically with the characterized ALDH1 from *Artemisia annua* and three contigs annotated as alcohol dehydrogenases grouped with the recently described ADH1 from *A. annua*. ALDH1 and ADH1 were characterized as part of the artemisinin biosynthesis. We have produced a comprehensive EST dataset for *T. laciniata* roots, which contains a large sample of the *T. laciniata* transcriptome. These transcriptome data provide the foundation for future research into the molecular basis for terpenoid biosynthesis in *Thapsia* and on the evolution of terpenoids in Apiaceae.

## 1. Introduction

Species of Apiaceae are widely distributed in the Mediterranean area and elsewhere, where they are often used commercially as spices or drugs due to the presence of desirable specialized metabolites [1]. The most characteristic constituents of Apiaceae are coumarins and sesquiterpene lactones [2–5], and also irregular terpenoids based on the thapsane carbon skeleton [6]. Several of the irregular terpenoids that have been found in Apiaceae have a structure that cannot be formed from the usual acyclic substrate molecules geranyl diphosphate (GPP, precursors to monoterpenoids), farnesyl diphosphate (FPP, precursors to sesquiterpenoids) and geranylgeranyl diphosphate (GGPP, precursors to diterpenoids) that are biosynthesized through head-tail fusion of IPP units, but are formed from tail-tail or head-head fused IPP units.

**Figure 1:**
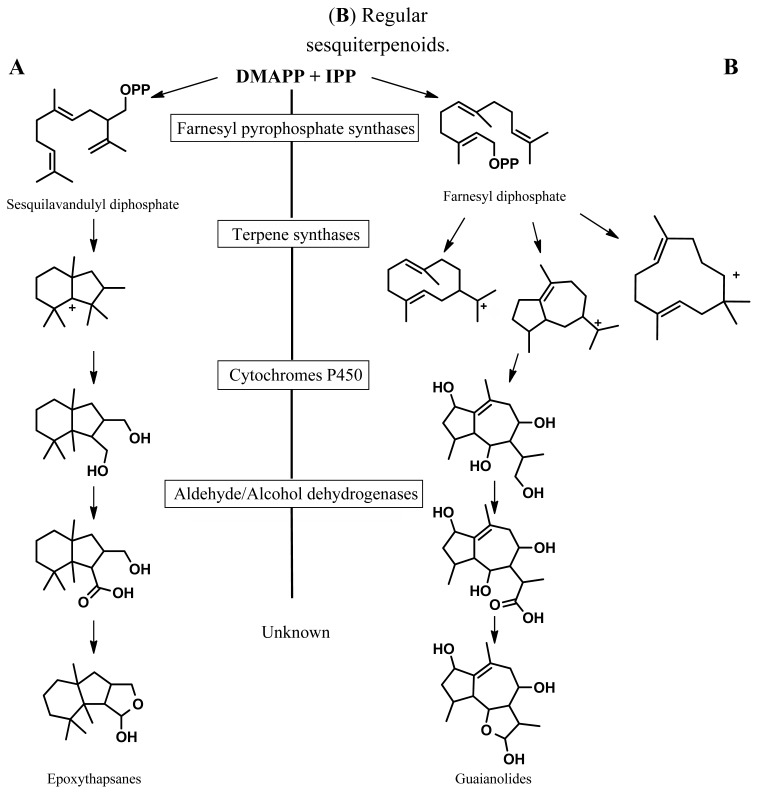
An illustration of the array of enzyme classes that are involved in the diversity of sesquiterpenoids in the Apiaceae. (**A**) Irregular sesquiterpenoids. (**B**) Regular sesquiterpenoids.

The species studied here, *Thapsia laciniata* Rouy (according to Weitzel *et al.* [7]), produces sesquiterpenoids based on the irregular thapsane carbon skeleton (Figure 1A), which has so far only been found in Apiaceae species. Thapsane-type sesquiterpenoids are thought to arise from the irregular terpene precursor, sesquilavandulyl diphosphate (SPP) [8], which itself is the product of an irregular farnesyl diphosphate synthase [9] that can perform one head-tail and one head-head fusion of the three IPP units giving rise to SPP. Only a few farnesyl diphosphate synthases making irregular precursors for the terpene synthases have been described so far, and this gene family therefore represents an unexploited area in the field of terpene biosynthesis and diversity [10,11].

In addition to this class of irregular terpenoids, *Thapsia laciniata* also contains regular sesquiterpenoids, such a guaienes, germacrenes, cadinenes, humulenes and hydroxylated derivatives thereof, such as oxy-caryophyllenes, and guaianolides (Figure 1B) [1,4,5]. The latter group includes thapsigargin, a major component and bioactive constituent of some *Thapsia* species. Thapsigargin induces apoptosis in mammalian cells via a high affinity inhibition of the Sarco/endoplasmic reticulum Ca^2+^ ATPase. Thapsigargins are currently valuable tools in the study of calcium homeostasis [6], and a derivative of a specific polyoxygenated sesquiterpene lactone from this class of compounds is currently undergoing clinical trials for the treatment of solid tumors [12]. While intensive chemotaxonomic studies have been performed on *Thapsia* sp. in order to investigate the distribution of these specific highly bioactive thapsigargins, the mechanism of biosynthesis of thapsanes and guaianolides has not been elucidated. However, probable biochemical precursors such as hydrocarbon or oxygenated sesquiterpenes have previously been identified in several *Thapsia* species [4–6,13], and recently the first putative enzyme in thapsigargin biosynthesis was characterized from *Thapsia garganica* [14]. Additionally, research into the biosynthesis of sesquiterpene lactones from Asteraceae species, despite differences in their basic stereochemistry, provides a basis from which one can infer the likely mechanism of Apiaceae-type sesquiterpene lactone biosynthesis. In particular, a great deal of work has been carried out to elucidate the biosynthetic pathway of the anti-malaria drug artemisinin from *Artemisia annua*, demonstrating the involvement of a regular FPP synthase, a sesquiterpene synthase, at least one cytochrome P450 from the 71 clade, and aldehyde and alcohol dehydrogenases [15]. Further work on *Lactuca sativa* has shown that the cytochromes P450 responsible for sesquiterpene hydroxylation are conserved, may be identified by phylogenetic analysis, but are likely to be promiscuous in their specificity for sesquiterpene substrates [16]. With this in mind, a transcriptomic analysis utilizing Illumina next-generation sequencing has provided an opportunity for identifying novel farnesyl pyrophosphate synthases, terpene synthases, cytochromes P450, and alcohol and aldehyde dehydrogenases that are likely to be involved in the biosynthesis of terpenoids in *Thapsia*. We chose *Thapsia laciniata* Rouy as a representative because of its unique chemical composition, including a diverse variety of oxygenated sesquiterpenoids [4,6]. The data from this study will be vital for the future biochemical elucidation of the biosynthetic pathways of both regular and irregular terpenoids, such as thapsigargins and thapsanes, respectively.

The genus *Thapsia* has been subjected to several revisions during the last century. The genus was initially divided into three species: *T. garganica* L., *T. maxima* Miller and *T. villosa* L. [17], with a fourth species, *T. transtagana* Brot., described more recently [18]. At least two other species, *T. minor* Hoffgg. and Link and *T. laciniata* Rouy have also been described [19]. A recent resurrection of the species led to a new and clearer classification [7]. This latest division has been adopted for this report, for which *T. laciniata* plants harvested in southern France were studied. Previous studies on the secondary metabolites of the genus *Thapsia* have shown clear variations between and within the species [6,20]. Only *T. minor* and *T. laciniata* contain the irregular terpenoids thapsanes, whereas the other *Thapsia* species contain highly decorated guaianolides, which are not found in *T. minor* and *T. laciniata* [6].

Despite the more than 4000 species in the Apiaceae family, nucleotide sequence data has thus far been generated for eight species only (based on publicly available data from NCBI in May 2012). *Daucus carota* L. (carrot) and the subspecies *sativus* show 43782 nucleotide and EST entries, and *Apium graveolens* L. and the variety *dulce* (celery) return 3528 nucleotide and EST entries that mostly arose from a single study [21]. *Centella asiatica* (L.) Urb. returns 4523 entries, and *Pimpinella brachycarpa* L., *Angelica sinensis* L., *Bupleurum chinense* DC and *B. kaoi* T. S. Liu each have several hundred nucleotide entries publicly available. The complete plastid genome of *D. carota* was published in 2006 [22], a BAC based library covering approximately 10% of the carrot genome was published in 2009 [23], as well as new transcriptome data in 2012 [24]. Thus, *D. carota* is currently the most thoroughly sequenced Apiaceae species, and although so far no species within Apiaceae has been fully sequenced and made publicly available, transcriptomes of several species are currently being sequenced, with *D. carota* and *B. chinense* being the only ones so far available at the SRA (sequence read archive) at NCBI [25].

Terpenes comprise the largest group of specialized plant metabolites with sesquiterpene lactones being a minor but highly valuable subgroup that are used in a range of industrial and medicinal applications [1]. As mentioned before, studies on the mechanism of biosynthesis of sesquiterpene lactones have so far been limited to the plant family Asteraceae [26–28], with a single recent study on sesquiterpene synthases from *T. garganica* that are thought to be the first step in the biosynthesis of thapsigargin [14] and possibly other guaianolides, but not thapsanes. An in-depth knowledge of the genomics of Apiaceae species, of which *T. laciniata* is a representative, will enable us to expand sesquiterpene lactone research to another plant family.

The objective of this study is to discover genes that encode for enzymes involved in the biosynthesis of terpenoids in Apiaceae, with a focus on sesquiterpenoids and lactones thereof, and the description of the related pathways. Our results demonstrate the potential of using next-generation sequencing to produce transcriptomic data from a medicinally interesting non-model species belonging to the under-investigated plant family Apiaceae. We use the transcriptome data to describe genes that are potentially involved in the biosynthesis of pharmaceutically relevant secondary metabolites from Apiaceae and identify potential novel gene targets to be cloned into heterologous hosts for production of economically interesting terpenoids.

## 2. Results and Discussion

### 2.1. Transcriptome Sequencing, *de novo* Assembly and General Metabolism

Sequencing was performed on RNA extracted from the roots of *T. laciniata*, utilizing two lanes of an Illumina GAIIx platform. A total of 66,776,746 high quality (HQ) reads with an average sequence length of 104.5 bp were obtained from 76,028,196 raw reads after the initial quality-filtering step. The HQ reads were assembled into 76,565 contigs with the use of the Trinity RNA-Seq assembly package [29], with a minimum length cut off set at 300 bp. 64.58 million of the 66.78 million reads were used in the assembly, with the rest not being assembled to larger contigs. The contigs had an N50 of 1261 bp, with a range of 300–16,683 bp and a total GC content of 41%.

The assembly for our sequencing reads was initially carried out with both the Trinity and Velvet assemblers. In general, multi-kmer assemblies are slightly poorer in terms of N50 values [30], but we found that the Trinity assembler was significantly better at resolving splice alternates in the dataset than Velvet (data not shown). The Trinity assembly also produced less duplicates or assembly chimeras, which are often introduced in a Velvet/Oases multi-run merging stage, hence generating a lower total number of long reads than the Velvet output. In addition, the ratio of reads recruited for assembly was greater in the Trinity assembly suggesting better coverage of the transcriptome. Since Trinity has this resolving power, its results were chosen for analysis.

The 76,565 assembled contigs obtained from the Trinity assembly were successively annotated with MAGPIE [31] through comparison with the sequences in major public protein databases (KEGG, the non-redundant NCBI Protein collection, the plant subset of Refseq, Interpro, and the NCBI Conserved Domain Database). A total of 47,408 contigs, accounting for 61.9% of the total contigs, were annotated. 52,101 were associated with Gene Ontology terms of which 43,816 contigs received high level hits (*E* < 10^−35^) (Tables S1 and S2: S1 is the summary of the annotation obtained from NCBI and S2 is the summary of the annotations obtained from InterPro including GO annotation).

The Illumina dataset can be used to substantially aid the discovery of novel genes involved in the general metabolism as well as specialized metabolism. When using *de novo* assembled transcriptomes for the identification of novel transcripts, it is first important to determine whether the depth of the sequencing is sufficient to enable the accurate and complete assembly of the majority of expressed transcripts. Using protein sequences of the well-described and highly conserved tricarboxylic acid (TCA) cycle as BLAST queries against our assembled contigs, we determined that transcripts encoding all of these enzymes could be identified (Table 1). Full length transcripts encoding all TCA cycle components enzymes were present in our assembly and were represented by between 7805 (in the case of Dihydrolipoyl dehydrogenase) and 194838 (in the case of isocitrate dehydrogenase) sequencing reads. This indicated that the depth of sequencing obtained from our Illumina sequencing of the *T. laciniata* transcriptome was sufficient for the assembly of complete transcripts, and also that the Trinity algorithm used for assembly was accurate. It is also important to note that little or no degradation of RNA had occurred prior to sequencing; otherwise, assembled contigs would not be full length.

Based on the search and annotation results, several definite sequence features could be used to identify the specific terpene synthases, cytochromes P450, aldehyde and possibly alcohol dehydrogenase involved in the modification of terpene skeletons. Candidate genes related to sesquiterpene backbone biosynthesis and similar to sesquiterpene synthases have been identified and will be discussed in detail, similarly for downstream enzymes involved in terpenoid biosynthesis such as cytochromes P450.

### 2.2. Candidate Genes for Farnesyl Diphosphate Synthase

Contig 3967 from our dataset was clearly identified as farnesyl diphosphate synthase (FPPS). In order to establish the suggested route to thapsanes, a synthase would be needed for the formation of sesquilavandulyl diphosphate (SPP) [9]. However, while the predicted protein encoded by contig 3967 exhibited 78% identity with its *Arabidopsis* orthologue (NP:199588), the second closest sequence in our dataset had only 25% identity. To investigate the possibility that a second FPPS-like gene (with potential SPP synthase activity) was present in the genome of *T. laciniata* but was not represented in the transcriptome dataset, we attempted to amplify other FPPS homologues from genomic DNA using degenerate primers. However, no additional FPPS sequences were retrieved. Thus, despite the structural similarity of FPP and SPP, it is likely that the enzyme involved in the formation of SPP has little sequence identity to FPPS. Previously, the only irregular prenyl synthase enzyme that has been biochemically characterized is chrysanthemyl diphosphate (CPP) synthase from Sagebrush [10,32], although the requirement of an enzyme with irregular farnesyl pyrophosphate synthase activity has been described [9]. Thus, the investigation of irregular prenyl diphosphate synthases is an interesting field for discovery of enzymes with novel and very chemically interesting functions.

### 2.3. Candidate Genes for Terpene Synthases

**Figure 2:**
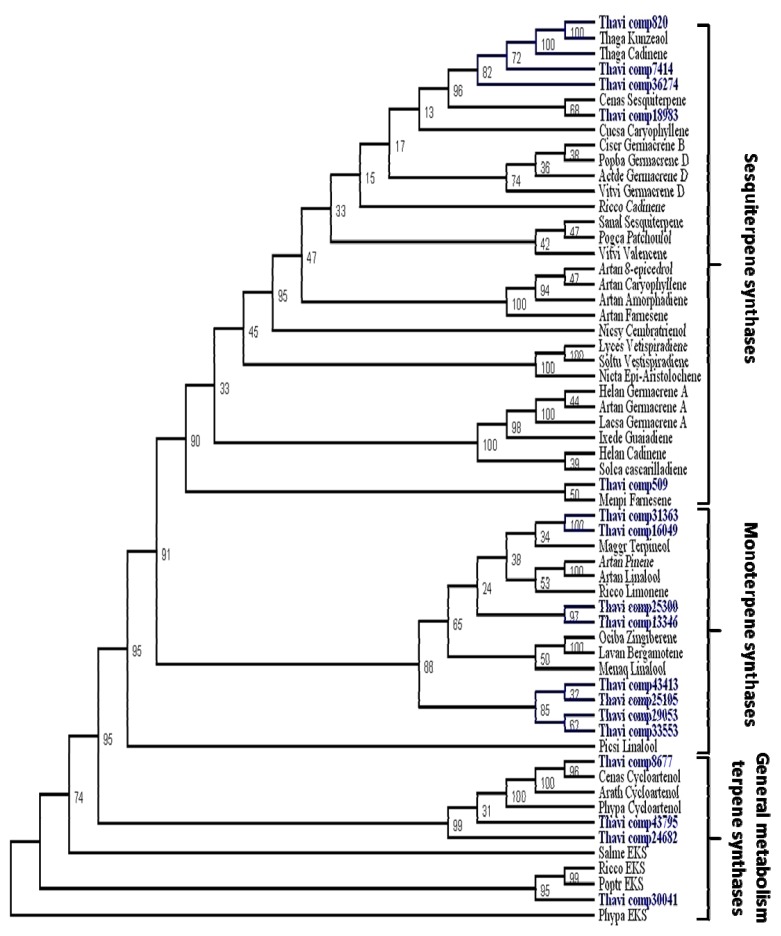
Phylogeny tree of terpene synthases. The tree illustrates the likely enzymatic function of 17 *T. laciniata* contigs. The figure clearly shows that comp820 is likely to be a Kunzeaol synthase, which is part of the guaianolide biosynthesis. The tree also shows a distribution of the *Thapsia laciniata* sequences throughout the three main clades of the tree. Phypa EKS (BAF61135, Ent-kaurene synthase, *Physcomitrella patens*), Poptr EKS (EEE88653.1, Ent-kaurene synthase, *Populus trichocarpa*), Ricco EKS (EEF28689.1, Ent-kaurene synthase, *Ricinus communis*), Arath Cycloartenol (AEC06032.1, cycloartenol synthase, *Arabidopsis thaliana*) Cenas Cycloartenol (AAS01524.1, cycloartenol synthase, *Centella asiatica*), Phypa Cycloartenol (Pp1s33_211V6.1 cosmoss, cycloartenol synthase, *Physcomitrella patens*), Nicsy Cembratrienol (ADI87448, cembratrienol synthase 3, *Nicotiana sylvestris*), Artan Linalool (AAF13356.1, (3R)-linalool synthase, *Artemisia annua*), Actde Germacrene D (AAX16121.1, germacrene-D synthase, *Actinidia deliciosa*) Menaq Linalool (AAL99381.1, linalool synthase, *Mentha aquatica*), Picsi Linalool (ADZ45502.1, (−)-linalool synthase, *Picea sitchensis*), Maggr Terpineol (ACC66282.1, α-terpineol synthase, *Magnolia grandiflora*), Artan Pinene (AF276072.1, (−)-beta-pinene synthase, *Artemisia annua*) Ixede Guaiadiene (AAL92481.1, guaiadiene synthase, *Ixeris dentata* var. *albiflora*), Artan Farnesene (AAX39387.1, (*E*)-β-farnesene synthase, *Artemisia annua*), Solca cascarilladiene (AAT72931.1, cascarilladiene synthase, *Solidago canadensis*), Artan Germacrene A (ABE03980.1, germacrene A synthase, *Artemisia annua*), Helan Cadinene (ACA33926.1, cadinene synthase, *Helianthus annuus*), Helan Germacrene A (ACA14463.1, germacrene A synthase 1, *Helianthus annuus*), Cucsa Caryophyllene (AAU05952.1, β-caryophyllene synthase, *Cucumis sativus*), Lacsa Germacrene A (AAM11626.1, Germacrene-A LTC1, *Lactuca sativa*), Popba Germacrene D (AAR99061.1, (−)-germacrene-D, *Populus trichocarpa* x *Populus deltoides*), Artan Caryophyllene (AAL79181.1, β-caryophyllene QHS1, *Artemisia annua*), Cenas Sesquiterpene (ABK63808.1, sesquiterpene cyclase, *Centella asiatica*), Pogca Patchoulol (AAS86323.1, patchoulol synthase, *Pogostemon cablin*), Salme EKS (ABV08817, emt-kaurene synthase, *Salvia miltiorrhiza*), Vitvi Germacrene D (AAS66357.1, (−)-germacrene D synthase, *Vitis vinifera*), Vitvi Valencene (AAS66358.1, (+)-valencene synthase, *Vitis vinifera*), Ciscr Germacrene B (ACF94469.1, Germacrene B synthase, *Cistus creticus subsp. Creticus*), Sanal Sesquiterpene (ADO87000.1, santalene synthase, *Santalum album*), Artan 8-epicedrol (AAF80333.1, 8-epicedrol synthase, *Artemisia annua*), Artan Amorphadiene (AF138959.1, amorpha-4,11-diene synthase, *Artemisia annua*), Ricco Cadinene (EEF39510.1, (+)-delta-cadinene synthase, *Ricinus communis*), Soltu Vestispiradiene (Q9XJ32.1, Vestipiradiene synthase, *Solanum tuberosum*), Thaga Cadinene (AFV09098.1, δ-cadinene synthase, *Thapsia garganica*), Thaga Kunzeaol (AFV09099.1, kunzeaol synthase, *Thapsia garganica*), Menpi Farnesene (AAB95209.1, farnesene synthase, *Mentha* x *piperita*), Nicta Epi-Aristolochene (3M02.A, 5-Epi-Aristolochene Synthase, *Nicotiana tabacum*), Ricco Limonene (XP:002533355.1, (*R*)-limonene synthase, *Ricinus communis*), Lavan Bergamotene (Q2XSC4.1, *E*-α-bergamotene synthase, *Lavandula angustifolia*), Ociba Zingiberene (Q5SBP4.1, α-zingiberene synthase, *Ocimum basilicum*), Lyces Vetispiradiene (AAG09949.1, Vetispiradiene synthase, *Solanum lycopersicum*).

Within our assembled contigs, 17 unique terpene synthase sequences were identified (Figure 2). Of these sequences, two could be identified as being involved in general metabolism, with contig 30,041 annotated as ent-kaurene synthase and contig 8677 as a cycloartenol synthase. The contigs 43,795 and 24,682 were not assigned putative functions based on the phylogenetic tree in Figure 2 due to incomplete sequences; however, they appear to group with triterpene synthases of general metabolism. The remaining 13 were located within clades corresponding to genes involved in the specialized metabolism of mono- and sesquiterpenoid biosynthesis. Of these, only contig 820 could be assigned a putative function because of its close homology to the recently described kunzeaol synthase from *T. garganica* [14]. To determine a definitive function, the remaining 12 contigs would need to be cloned, expressed and biochemically characterized in order to determine their terpene products. Nevertheless, phylogenetic analysis indicates that contigs 7414, 36274, 18983, and 509 are likely to produce sesquiterpene backbones, while contigs 13346, 16049, 25105, 25300, 29053, 31363, 33553, and 43413 are all likely to be involved in monoterpenoid biosynthesis (Figure 2).

The number of terpene synthases identified here, 8 monoterpene synthases and 5 sesquiterpene synthases, is slightly larger than the number found in *Arabidopsis*, where 4 mono and 3 sesquiterpene synthases were identified [33], while *Artemisia annua* contains at least 4 mono and 6 sesquiterpene synthases (based on a NCBI search of published sequences). This indicates that the number found in *Thapsia* corresponds well to what can be expected of mono and sesquiterpene synthases in one plant species. The relatively large number of terpene synthases found in *T. laciniata* roots is in contrast to *T. garganica* roots, where only two sesquiterpene synthases were found in the transcriptome in a recent study [14]. This is in agreement with the larger chemical diversity of complex terpenoids found in the roots of *T. laciniata*, which is in accordance with several studies on the chemistry of *Thapsia* plants [5,6]. The sesquiterpene synthases described here are currently undergoing biochemical characterization.

**Figure 3:**
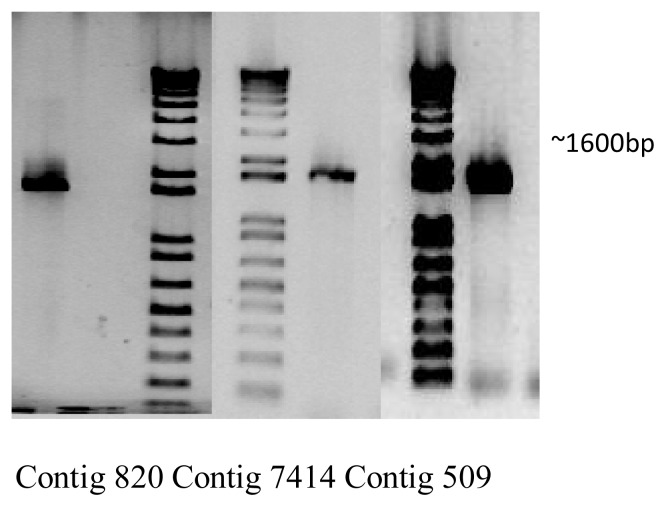
Agarose gel electrophoresis of PCR products amplified using specific primers for the genes represented by contigs 820, 7414 and 509, with predicted sizes of 1600 bp.

In order to confirm that the assembled contigs generated using the Trinity assembler were accurate, we designed primers to anneal around the predicted start and stop codons of contigs 820, 7414 and 509, representing three predicted full-length terpene synthases with expected lengths of approximately 1600 bp. All three genes could be readily amplified from cDNA synthesized from RNA isolated from a different *T. laciniata* plant than was originally sequenced (Figure 3). Subsequent sequencing of these nucleotides demonstrated that the sequences were exactly as predicted by the contig assembly, demonstrating that our contig collection is likely to be an accurate representation of nucleotide sequences. The three genes amplified are currently undergoing biochemical characterization.

### 2.4. Candidate Genes for Cytochromes P450 of the CYP71 Superfamily

Each sequence of a minimal dataset of plant cytochromes P450, containing one member of each subfamily known from plants [34], was used to perform a BLAST search against the Trinity *T. laciniata* transcriptome assembly. In this way, 67 contigs were annotated and assigned a putative CYP number based on homology to a single member of the minimal plant P450 collection (see Table S3). Almost all of the sequences appeared to be full length, containing nucleotides corresponding to the start and stop codons, with only 4 lacking some additional sequence information. A number of biosynthetic genes from general metabolism, including orthologous of the sterol 14α-demethylase enzyme CYP51G1, ent-kaurene oxidase CYP701A, and steroid 23-alpha-hydroxylase CYP90A were found in the list of annotated *T. laciniata* cytochromes P450. Of the 67 cytochrome P450 contigs, 12 could be assigned to the CYP71 family, 4 in the CYP76 family and 2 in the CYP83 family. These families are part of the CYP71 clade and thus of special interest, since members of this clade have been attributed to specialized terpenoid metabolism [16,27,35]. The sequences assigned the CYP numbers CYP71D183 (contig 1645) and CYP71D319 (contig 15,003) show close homology to CYP71AV1 and CYP71BL1+2 that are known to be involved in the oxidation of the sesquiterpenes amorphadiene and germacrene A, respectively [16,27]. These sequences are therefore of high interest in relation to guaianolide biosynthesis since numerous hydroxylations of the guaiene-type backbones are needed for the complete biosynthesis of the highly modified guaianolides found in *T. laciniata*. Any of the 18 genes in the 71, 76 and 83 families could potentially be involved in the biosynthesis of sesquiterpene lactones in *Thapsia*.

In a recent study, a transcriptome dataset for *B. chinense* was obtained using 454 sequencing, with saponin biosynthesis as the target enzymes (a triterpene) [25]. In this study, 44 cytochromes P450 were found, with 4 being in the CYP71 clade, thus the number found in *Thapsia* is significantly higher, most likely due to the increased depth of the sequencing. With the CYP71 clan by far being the largest of the 11 land plant cytochrome P450 clans, encompassing more than half of all the plant cytochromes P450, extensive blooming of subfamilies within this clan appears to have taken place in a species-specific manner [36]. While giving rise to many interesting genes with diverse functions, this also makes cross-species comparison much more difficult with regard to functional annotation based on sequence similarity.

With more than 240 cytochromes P450 in *Arabidopsis* [35]*,* it is highly likely that not all cytochromes P450 from *T. laciniata* were discovered in our root-specific sequencing due to the different expression profiles of the various enzymes.

### 2.5. Candidate Genes for Aldehyde Dehydrogenases Involved in Sesquiterpenoid Biosynthesis

From the plant *Artemisia annua,* the aldehyde dehydrogenase ALDH1 was recently biochemically characterized and shown to catalyze the oxidation of dihydroartemisinic aldehylde into dihydroartemisinic acid [37]. Thus, homologues of this enzyme could potentially be involved in the modification of oxygenated sesquiterpenes that are required for formation of a lactone moiety (Figure 1). A survey on NCBI using the ALDH1 protein sequence (gb: ACR61719.1) using BLASTp, followed by Neighbour joining tree of this (data not shown), showed that ALDH1 formed a small clade, along with one other uncharacterized aldehyde dehydrogenase from *Saussurea medusa* (gb: AAT44126.1). This sub-clade formed part of the more universal F2 clade of cytosolic plant ALDHs [37]. The sequences of these two proteins were therefore used to search in our transcript data for sequence encoding similar enzymes that could be involved in terpenoid biosynthesis.

**Figure 4:**
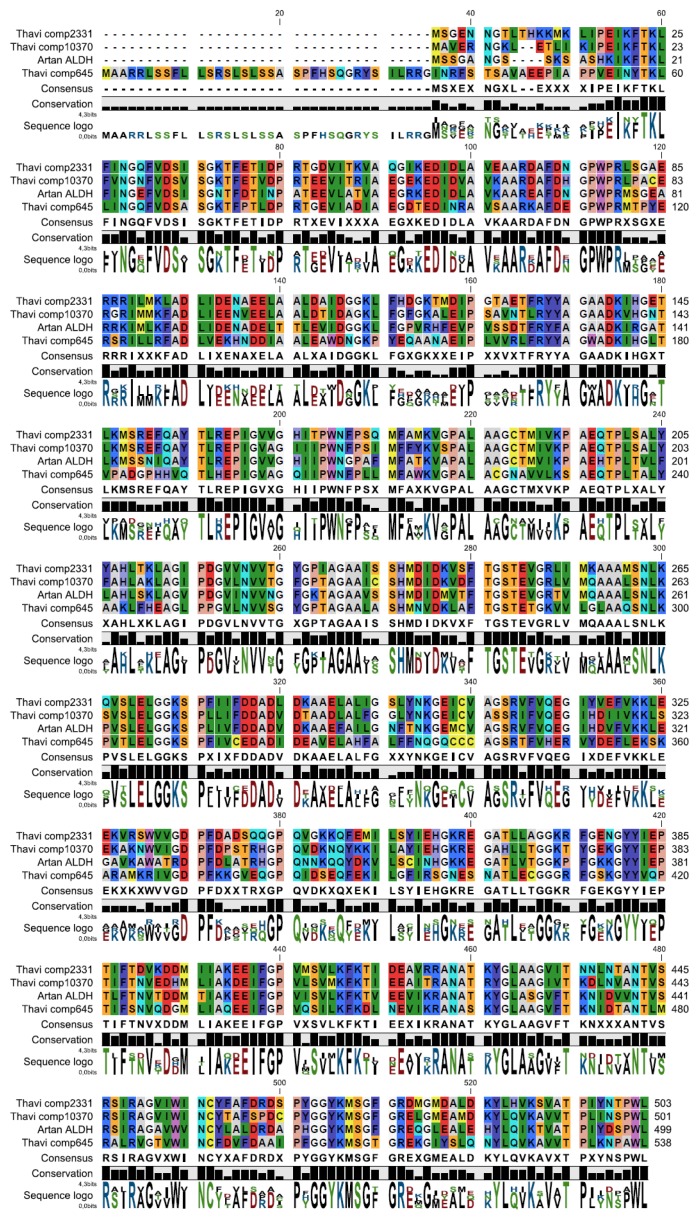
Alignment of *A. annua* ALDH and three *T. laciniata* contigs. Alignment of proteins encoded by contigs 645, 2331 and 10370 with *A. annua* ALDH1 (gb: ACR61719), illustrating the high amino acid sequence similarity. The consensus sequence is shown below the alignment and the sequence logo is at the bottom. Contig 645 contains a mitochondrial targeting sequence at the *N*-terminus.

In this clade, we find two *T. laciniata* sequences, contig 2331 and contig 10,370; these two transcripts are of major interest in the future search for genes potentially involved in sesquiterpene lactone biosynthesis. Contigs 2331 and 10,370 encode proteins that exhibit 69% amino acid identity with *A. annua* ALDH1, and both group in the same sub-clade of F2 ALDHs. Contig 645 encodes a protein that groups in the related clade of mitochondrial F2 ALDHs and contains a targeting peptide at the *N*-terminus (Figure 4). Other ALDH encoding transcripts in our *T. laciniata* transcriptome include contig 155 in the F2 mitochondrial family, contig 24,667 in the 3H1/3I1 family, and contig 8241 in the plant specific 22A1 family (data not shown). Three contigs, 23,206, 27,004, and 20,996, did not fall into a described clade. Other than contigs 2331 and 10,370 (Figure 4), no other *T. laciniata* ALDHs exhibited greater than 52% sequence identity with *A. annua* ALDH1. The family annotations described here have been adopted from previous published results [38].

### 2.6. Candidate Genes for Alcohol Dehydrogenases Involved in Sesquiterpenoid Biosynthesis

**Figure 5:**
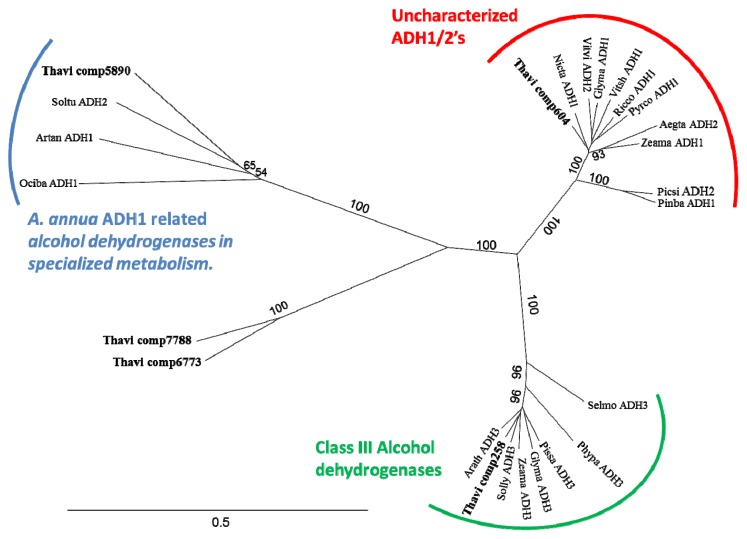
Phylogeny of Alcohol dehydrogenase. The tree shows that one *T. laciniata* ADH is found in the Class III clade as well as the uncharacterized but widely-spread ADH1-2 clade. In addition, the clade including *A. annua* ADH1 includes 4 *Thapsia* contigs. Aegta ADH2 (ABL74260, *Aegilops tauschii*), Arath ADH3 (CAA57973, *Arabidopsis thaliana*), Artan ADH1 (AEI16475.1, *Artemisia annua*), Glyma ADH1 (XP:003523232, *Glycine max*), Glyma ADH3 (XP:003521319.1, *Glycine max*), Nicta ADH1 (AAT40104, *Nicotiana tabacum*), Ociba ADH1 (AAX83109, *Ocimum basilicum*), Phypa ADH3 (XP:001764007, *Physcomitrella patens* subsp*. patens*), Picsi ADH2 (ABK24000, *Picea sitchensis*), Pinba ADH1 (AAC49540, *Pinus banksiana*), Pissa ADH3 (P80572.1, *Pisum sativum*), Pyrco ADH1 (AEL75211, *Pyrus communis*), Ricco ADH1 (XP:002534157, *Ricinus communis*), Selmo ADH3 (XP:002973821, *Selaginella moellendorffii*), Solly ADH3 (NP:001238796, *Solanum lycopersicum*), Soltu ADH2 (CAA63093, *Solanum tuberosum*), Vitsh ADH1 (ADF80913, *Vitis shuttleworthii*), Vitvi ADH2 (AF194174.1, *Vitis vinifera*), Vitvi ADH6, AF195866.1, *Vitis vinifera*), Zeama ADH1 (NP:001105409, *Zea mays*), Zeama ADH3 (ACG32791, *Zea mays*).

From the plant *Artemisia annua* the aldehyde dehydrogenase ADH1 (gb: AEI16475) was recently biochemically characterized and showed to be involved in the oxidation of artemisinic alcohol into artemisinic aldehyde [39,40]. A survey of the NCBI non-redundant protein database using the ADH1 protein sequences with BLASTP, followed by Neighbor-joining tree of this (data not shown), showed that *A. annua* ADH1 grouped with a number of uncharacterized alcohol dehydrogenases. These formed a unique clade clearly distinct from the class 3 alcohol dehydrogenase enzymes found throughout eukaryotes known as ADH3 or glutathione dependent formaldehyde dehydrogenases (FALDH, EC 1.2.1.1). 42 *Thapsia* contigs shared sequence similarity with ADH1 from *A. annua* (Table S1). Of these, 6 were full length and additionally another three were found to be almost full length (estimated to be above 90% of the full length). The contig 6804 was annotated as sorbitol dehydrogenase and was not added to phylogenetic tree. In a phylogenetic tree including a selection of ADH1, ADH2 and ADH3 family enzymes, and the 5 *T. laciniata* transcripts with the highest similarity to *A. annua* ADH1, contig 5890 clustered closely with *A. annua* ADH1 while contigs 6773 and 7788 branched off the same clade (Figure 5). Contig 258 clustered with the Class III ADHs and contig 604 clustered in a well-defined clade of uncharacterized ADHs distinct from *A. annua* ADH1.

## 3. Experimental Section

### 3.1. Plant Material and RNA Extraction

*Thapsia laciniata* Rouy roots were collected in June 2010 just west of Cannes, France (GPS: 43.540958, 6.816158). Whole living healthy flowering plants were removed from the ground and transported in soil back to Denmark, over a period of approximately three days, where they were snap frozen in liquid nitrogen and stored at −80 °C until RNA extraction. A voucher specimen (HTS 2010-01) has been deposited at the KU-LIFE herbarium (CP) and was the basis of this study.

### 3.2. cDNA Library Construction and Sequencing

Total RNA was isolated from *T. laciniata* roots using the CTAB method [41]. For the isolation of RNA one root (10 cm long and *ca.* 20 g) was taken out of the freezer, cleaned in sterile water and crushed into pieces under liquid N_2_. Approximately 1 g of tissue, centrally located in the root, was subjected to RNA extraction as described previously [41], yielding 300 μg total RNA as determined by nano-drop. RNA integrity was initially confirmed by agarose gel electrophoresis and the visualization of intact ribosomal RNA bands. Subsequent RNA quality control was carried out on a 2100 Bioanalyzer (Agilent Technologies, Hørsholm, Denmark) and each sample received an RNA integrity numbers (RIN) of greater than 8.5. The Poly A selection, preparation of cDNA, ligation of adapters, cluster formation and sequencing was performed at the McGill University and Genome Quebec Innovation Centre according to the manufacturer’s recommendation and using standard Illumina kits. Size selection was performed using Sage Science’s Pippin Prep DNA size selection system (a band was eluted at around 350–400 bp, that size includes the adapters which are about 120 bp). The sequencing was done on an Illumina GAIIx instrument for a paired-end run of 2x108 cycles. The raw reads have been uploaded to the Sequence Read Archive (SRA) at NCBI with the accession number SRP019808.

### 3.3. Sequence Analysis and Assembly

Raw Illumina sequence data were preprocessed before assembly. FastQC (version 0.7.2) [42] was run to obtain sequence statistics and to determine cleaning parameters. Removal of adapter contamination was first performed using Cutadapt [43]. In-house Perl5 scripts were subsequently used for windowed quality clipping at a quality score cutoff of 25, a 12 bp 5′ trimming to reduce bias associated with random priming during library preparation and finally a 35 bp minimum length filter (removal was carried out on read pairs).

The Trinity *de novo* RNA-Seq assembler (Release 19 May 2011) was used to generate the full transcriptome assembly. The following parameters were set for Trinity module “Butterfly”; graph compaction option: edge-thr = 0.26, path extension mode = compatible_path_extention, min_contig_ length = 300, paired_fragment_length = 270 (50 bp + estimated median fragment size of readset). Standard settings were used otherwise.

CLC Genomics 4.8 (48014) provided by CLC bio (www.clcbio.com) was used as sequence handling program in general, and the assembler provided with the program, though designed for mapping use, was used with the following settings: Similarity = 0.8, Length fraction = 0.5, Insertion cost = 3, Deletion cost = 3, Mismatch cost = 2, Min distance = 180, Max distance = 300, and with a contig cutoff at 200.

### 3.4. Mapping, Functional Annotation and Pathway Assignments

Based on the assembly statistics, only the Trinity assembly was taken forward. To obtain a wide base of evidence for determining function, the assembly set was annotated with MAGPIE through protein level similarity searches against NCBI’s non-redundant (nr) database and the viridiplantae subset of NCBI RefSeq using BLASTX (*E*-value < 10^−3^) and against the NCBI Conserved Doman Database (CDD) using RPS-BLAST (*E*-value < 10^−2^). The InterPro suite of protein family and domain databases was also queried by Hidden Markov model (HMM) searches performed with HMMER [44] (*E*-value < 10^−10^). GO terms were associated to individual transcripts based GIDs extracted from their respective search hits. High level annotations were determined as BLASTX hits with *E*-value < 10^−35^, RPS-BLAST hits with *E*-value < 10^−25^, and finally HMM results with *E*-value < 10^−20^ as well as a percentage similarity of at least 65%. These high level annotations were subsequently used in mapping and assigning EC values to their respective contigs. The weighed sum of annotations for each contig was summarized to give a putative functional description [45–47].

All hits annotated to with text P450 or Cytochrome were blasted against a set Minimal set of Plant cytochromes P450 and the cytochromes P450 were then annotated to their specific family; full length cytochromes P450 obtained a CYP number from David Nelson (The University of Tennessee Health Science Center, Memphis, TN, USA) [36].

To determine metabolic pathways, the Kyoto Encyclopedia of Genes and Genomes (KEGG) mapping was used [47]. To obtain full pathway annotation and to identify the functional hierarchies, all contigs from the Trinity assembly were submitted to the KEGG Automatic Annotation Server (KAAS) [45], and the single-directional best hit information method was selected and listed in Table S1. KAAS annotates every submitted sequence with KEGG orthology (KO) identifiers, which represents an orthologous group of genes directly linked to an object in the KEGG pathways and BRITE functional hierarchy [45,46] and thus incorporates different types of relationships that exist in biological systems (*i.e.*, genetic and environmental information processing, cellular processes, and organismal systems).

### 3.5. Alignment and Tree Building

All alignments used for the construction of phylogenetic trees were performed with the in-built Muscle alignment in Geneious (version 6.0.4; Biomatters Ltd.: Auckland, New Zealand, 2012). The alignment was performed as a free-end gap, and the computational alignment was followed by a hand sorting.

All trees were constructed in Geneious (version 6.0.4) using the LG model of amino acid substitution [48]. Bootstrap information was added to all trees with 100 repetitions.

### 3.6. Amplification and Sequencing of Representative Full-Length Terpene Synthases

From plant material collected as described in section 3.1, RNA was extracted and cDNA synthesized as described in section 3.2. Three primer sets for the three full length terpene synthases, contigs 820, 509 and 7414, were designed.

For contig 820, Forward: CTGCGGCCGCATGGCTGTGTATGTTAAC and reverse: CTAGATCTTTATGCTGGAATGGGATT.

For contig 509, Forward: CAGCGGCCGCATGGGCAGCCCGTCTCG and reverse: GCGAGCTCTCATATTGGTATGGGATCCATAAG.

For contig 7414, Forward: CAGCGGCCGCATGGCTATGTGTGTTAATTC and reverse: CTGAGCTCTTATACAGGAACAGGGTCC.

The PCR amplification was performed under the following conditions; PCR cycling: 96 °C (10 min); 30 cycles of 96 °C (30 s), 50 °C (30 s), 72 °C (1.5 min); 72 °C (10 min), and PCR-mix was composed of 2 μL of 10× X7-buffer, 0.5 μL of 10 mM dNTP solution, 1 μL of each primer solution, 0.2 μL of X7-Polymerase (Pfu X7) [49], 1.5 μL of the cDNA from *T. laciniata* root, and water up to 20 μL. The electrophoresis was performed as 3 μL of each sample was loaded in a 1% agarose gel and visualized with xylenol orange (150 V for 25 min). Nucleotide sequencing was carried out by MWG Eurofins.

## 4. Conclusions

Nucleotide sequences for plants from the Apiaceae family have previously only been available, in limited numbers, for *D. carota* and *B. chinense.* Here, we present a large sequence collection from a third Apiaceae species, *T. laciniata*, consisting of more than 76,000 contigs. Using Illumina high-throughput sequencing, we have produced a comprehensive EST dataset for *T. laciniata* roots, which contains a large sample of the *T. laciniata* transcriptome. Five sesquiterpene synthases and 16 cytochromes P450 in the CYP71 clade have been described here, along with candidates from the ALDH and ADH enzyme families, and will provide the foundation for future research aimed at uncovering the molecular basis for terpenoid biosynthesis in *Thapsia* and on the evolution of terpenoid biosynthesis in the Apiaceae.

## Figures and Tables

**Table 1:** *T. laciniata* tricarboxylic acid cycle (TCA) encoding transcripts. Individual components of the TCA cycle were identified by using corresponding Arabidopsis enzymes as queries in a tBLASTn search of the assembled *T. laciniata* contigs. The identified contigs are listed with their amino acid identity to their *Arabidopsis orthologues* shown in parenthesis. The number of reads from which the contigs were assembled, giving an absolute expression value, is shown in the fourth column.

TCA cycle enzyme	Arabidopsis query	Contig (identity)	Reads
Citrate synthase	NP_850415	Comp7488 (84%)	12696
Aconitase	NP_567763	Comp1618 (85%)	46037
Isocitrate dehydrogenase	NP_175836	Comp214 (85%)	194838
2-oxoglutarate dehydrogenase	NP_191101	Comp359 (85%)	162552
Dihydrolipoyllysine succinyltransferase	NP_200318	Comp2028 (71%)	35422
Dihydrolipoyl dehydrogenase	NP_567487	Comp12031 (84%)	7805
Succinyl-CoA synthetase	NP_001119263	Comp3388 (90%)	28993
Succinate dehydrogenase flavoprotein subunit	NP_201477	Comp2812 (94%)	29604
Succinate dehydrogenase iron-sulfur subnunit	NP_001118718	Comp3447 (76%)	21231
Fumarase	NP_001078075	Comp8438 (87%)	13149
Malate dehydrogenase	NP_190336	Comp4342 (77%)	19283
